# A Case of Dibotrocephalus Latus Infection1Read at the fortieth annual meeting of the Medical Association of Central New York, held at Rochester. October 15, 1907.

**Published:** 1908-04

**Authors:** Charles O. Boswell

**Affiliations:** Rochester, N. Y.


### A Case of Dibotrocephalus Latus Infection[1]

By CHARLES O. BOSWELL, M. D., Rochester, N. Y.

THE opportunity of presenting this case is due to the courtesy of Dr. Milton Chapman, one of the laboratory staff of the Rochester City Hospital, through whom the patient was admitted to the hospital ward, he having seen the woman previous to her admission.

---

1. Read at the fortieth annual meeting of the Medical Association of Central New York, held at Rochester, October 15, 1907.

CASE.—Mrs. L. V. Russian Jew, aged 19; admitted to the Rochester City Hospital, May 20, 1907. The history was obtained with difficulty through an interpreter.

She claims she was always well until a few weeks ago. Was married some six months before entering hospital. About six weeks previous to admission, while on shipboard, thought she had miscarried. Two weeks previous to admission complained of abdominal pain, flowing, some pelvic pain, worse on urination, some nausea and vomiting, some diarrhea with a little blood in the stools.

*General appearance.*—Fairly well nourished, rather sallow, tongue coated; heart accentuated second sound at the mitral valve. Liver somewhat enlarged, palpable below free border of ribs. Left Fallopian tube enlarged. On admission to ward pulse, 120; temp., 99°; respiration, 20. Evidence of ischiorectal abscess present. A blood examination was made on May 26, with the following result:

| | |
|---|---|
| Red Cells | 4,270,000 |
| White Cells | 9,600 |
| Hemoglobin | 95% |

Differential count:

| | | Per cent |
|---|---|---|
| Small Monoblasts | 38 | 12.1 |
| Large Monoblasts | 21 | 6.6 |
| Polymorphonuclears | 240 | 76.4 |
| Mast Cells | 2 | .6 |
| Eosins | 13 | 4.1 |
| | 314 | 99.9 |

Red cells slightly pale.

On examination of feces large numbers of eggs of an intestinal parasite were found. During the evening of June 1, she was given 1 dram of oleresin of aspidium, in combination with 1 ounce of sulphate of magnesia. About two hours later a large tapeworm which on examination proved to be a dibotrocephalus latus was expelled. This worm was about eight meters in length, but the head was not certainly identified.

Another blood count on June 3, gave this result: Red cells, 4,275,000; white cells, 10,660; hemoglobin, 90%.

Differential count:

| | | Per cent |
|---|---|---|
| Small Monoblasts | 60 | 20.0 |
| Large Monoblasts | 20 | 6.6 |
| Polymorphonuclears | 207 | 69.0 |
| Mast Cells | 1 | .3 |
| Eosins | 12 | 4.0 |
| | 300 | 99.9 |

The patient was shortly afterward discharged and Dr. Chapman reports that she seems in good health at present.

This tapeworm is extremely rare in the United States. There having been but thirty cases reported according to Stiles, quoted from Osler's System of Medicine. It is, however, very common in some parts of Europe, especially in those regions where raw or partially cooked fish form no inconsiderable part of the dietary. Accordingly we find it most commonly about the Baltic, Provinces of Prussia and Russia. Around the Swiss lakes and in parts of Ireland, so that it has also been called the Irish tapeworm. All reported cases in the United States have occurred in immigrants from these regions.

This worm is the largest of the tapeworms. It has a length of from 8 to 10 meters and presents as many as 3,000 to 4,000 segments, each of which are 25x4 mm. long by 8x14 mm. broad. The head is almond shaped, has no hooks but two longitudinal grooves believed to represent suckers. The neck is very short and narrow. The uterus is distinctly rosette shaped, this radiating structure serving to differentiate it clearly from the transverse arrangement of this organ in the Tenia solium and T. saginata Abortive and misformed segments are so common as to be considered a distinctive feature of this worm. Usually but a single worm is present, although at times a multiple infection occurs, as many as five different parasites having been determined. In these cases of multiple infection the different worms are of a much shorter length as a rule; although in Willson's case, which will be quoted later, he recovered at least two worms, neither of which could be less than 35 feet long. Simon is authority for a case in which 100 different worms were present at once. This worm has been found capable of reproducing as many as twenty to thirty segments per day.

The eggs are oval, 06-07 mm. long x .045 mm. broad. have a brownish envelope with a little lid at the anterior end. The larva which are round, and ciliated and provided with six hooklets, pass out through this lid as soon as the eggs have been deposited in water. They soon develop into the measle stage or piceroid, a small worm, which when eaten by certain fish, especially the pike, perch, and salmon, pass from the intestine of the fish into its muscles. When such infected fish are eaten by man the adult worm develops in the human intestine. No cysticercus stage has been noted in man. Dogs and cats and even foxes, are known to be occasional hosts of this parasite.

*Symptoms.*—There are no symptoms peculiar to this form of worm other than those of any tapeworm infection, except that it is more liable than any other cestode to produce a severe anemia. This point will be discussed later.

*Diagnosis.*—This, of course, must rest chiefly upon recognising the segments which may be passed at stool. Strümpell states that this occurs most often in the spring and fall. but no other authorities make such a claim. The eggs are usually abundantly present in the stool and if recognised make the diagnosis certain, but this is not an easy matter to the unfamiliar person. The practical point in diagnosis is that all seeming cases of pernicious anemia should have an examination of the stool made in order to exclude not only this parasite, but several others whose presence may also profoundly influence the blood picture, an anthelmintic being first given.

*Duration of infection.*—This worm may live in the intestinal tract for a long time. Ward reports one case where the worm was known to be present for thirty-five years, and in Willson's case this was known to be present at least fifteen years.

*Historical sketch.*—This worm was first described in 1603, but very early medical writings distinguish a broad tapeworm. Sommer and Landor's first worked out its anatomy in 1872. The first recorded case in this country, as far as known, was reported in 1879 by Walker and Leidy. An excellent article in the *American Journal of the Medical Sciences,* 1902, by Willson, of Philadelphia, gives the best available description of this infection and I will take the liberty of referring to it very fully.

He reports a case treated by him at the Pennsylvania Hospital in 1899. The patient was an immigrant from the Baltic provinces. From this case he recovered what seemed to him to indicate probably three worms, but only two heads were determined. In all there were about 27 metres of worm recovered. The patient's blood never showed any marked anemia. but there and of the eosinophiles, but the exact figures are not stated. (This was a proportionate increase both of the polymorphonuclears and of the oesinophiles, but the exact figures are not stated. (This is the case where the infection was known to be present for fifteen years).

*Anemia.*—As has been mentioned there is in some cases of this infection a very pronounced anemia which may exactly simulate both in absolute values and morphologically the blood picture considered representative of pernicious anemia. Even the post mortem changes of this latter disease has been noted. Emerson of Johns Hopkins Hospital in his textbook on Clinical Diagnosis gives as the average red count 1,300,000 with limits of 3,950,000 to 2,950,000. The majority of the nucleated red cells are of the variety known as megaloblasts. All writers agree that the blood picture improves very rapidly when even a part of the worm is expelled.

Naturally there has been considerable discussion as to why in some cases there should be such a tremendous blood destruction while, again, other cases show no appreciable change. Schapiro in a number of the *Zeitschrift für Klinische Medizin for* 1886, held that some toxic substance existed in the worm and was responsible for the hemolysis. It has even been suggested that this hypothetical toxin exerted its effect primarily on the bone marrow. Emerson also holds this view. Schauman and Tollquist extracted from the body of one of these worms a substance which when infected into dogs had a marked hemolytic effect.

The influence of hemorrhage in producing this anemia may be briefly discussed. The amount of blood in the stools is rarely present in any considerable amount, even if present at all. Of course, repeated small and concealed hemorrhages may be occurring but the hemorrhagic anemias are of the chlorotic type and the nucleated red cells are normoblasts, not megaloblasts. One feature of the blood picture worthy of mention is that an eosinophilia is not seen in these cases, whereas it is not uncommon in other intestinal infections by the beef and fish tapeworm, and especially in cases of hookworm infection. Ward explains this by assuming that the dibotrocephalus has not a chemiotactic influence for eosinophiles.

The claims of the followers of the toxin theory for these anemias are open to many criticisms. The lack of anemia in many cases and the absolute independence of this feature on the length or plurality of the infection, has lead to many attempted explanations. Dehio assumed that in these cases the worm must be dead or diseased, but this claim has not been established, and would be very difficult to prove. Willson would account for the anemia in this way. The intestines have a certain function in preparing substances for the use of the blood-making organs. Now, either the presence of this worm, or the nervous shock of the knowledge of its presence, interferes with this function; as a result the manufacture of substances usually produced for this end by the glands of the digestive tract ceases. Again, a certain toxic substance always present in the intestinal tract takes on a greater virulence as the result of the growth of the worm, and this works deleteriously on the blood-forming organs, or, again, the worm takes to itself a great deal of the nutriment meant for its host. He argues in favor of this view—that the prompt recovery of the patient's blood as soon as even part of the worm has been expelled, is incompatible with the length of time that would be needed to enable it to recover from an absorption of a toxin, were one formed by the worm.

This theory states, in other words, that the worm acts in part mechanically and in part also a true parasite, in that it consumes substances needful for its host. When the parasite has been expelled the intestines reassume their proper function. It does not seem that this theory explains very much better than some previous ones. Is it allowable to assume that some individuals may possess as it were a partial or even a complete immunity to such parasites? Of course, there are plenty of analogies drawn from other diseases due to living agents which can be called upon to strengthen such a view but reasoning from analogies is particularly fallacious in medicine. Another objection is, that while Willson states that recovery even after partial expulsion of the worm is too prompt to allow of the recovery from a toxemia if such is assumed to have been established he does not hesitate to assume that the increased virulence of some unknown and problematic intestinal metabolism which he has in mind, is conveniently and promptly done away with. Is it not just as reasonable to dispute both promises as to allow of the one fitting his view? Certainly no intestinal product which can produce the tremendous alteration in the blood seen in pernicious anemia has yet been found.

*Prophylaxis.*—With the steady increase of immigrants into this country from infected regions, bringing not only the means of further propagating this disease but also their unhygienic modes of life, made even worse by their close segregation in our larger cities, it is very probable that this disease will soon cease to be rare in the United States unless it be recognised and proper care in the disposal of feces be assured.

41 Gibbs Street.

